# *TGFβ1* Polymorphisms Predict Distant Metastasis–Free Survival in Patients with Inoperable Non-Small-Cell Lung Cancer after Definitive Radiotherapy

**DOI:** 10.1371/journal.pone.0065659

**Published:** 2013-06-19

**Authors:** Xianglin Yuan, Qingyi Wei, Ritsuko Komaki, Zhensheng Liu, Ju Yang, Susan L. Tucker, Ting Xu, John V. Heymach, Charles Lu, James D. Cox, Zhongxing Liao

**Affiliations:** 1 Department of Oncology, Tongji Hospital, Tongji Medical College, Huazhong University of Science and Technology, Wuhan, China; 2 Department of Epidemiology, The University of Texas MD Anderson Cancer Center, Houston, Texas, United States of America; 3 Department of Radiation Oncology, The University of Texas MD Anderson Cancer Center, Houston, Texas, United States of America; 4 Department of Bioinformatics and Computational Biology, The University of Texas MD Anderson Cancer Center, Houston, Texas, United States of America; 5 Department of Thoracic/Head and Neck Medical Oncology, The University of Texas MD Anderson Cancer Center, Houston, Texas, United States of America; University of Kentucky College of Medicine, United States of America

## Abstract

**Purpose:**

Transforming growth factor (TGF) -β1 signaling is involved in cancer-cell metastasis. We investigated whether single nucleotide polymorphisms (SNPs) at *TGFβ1* were associated with overall survival (OS) and distant metastasis-free survival (DMFS) in patients with non-small cell lung cancer (NSCLC) treated with definitive radiotherapy, with or without chemotherapy.

**Methods:**

We genotyped *TGFβ1* SNPs at rs1800469 (C–509T), rs1800471 (G915C), and rs1982073 (T+29C) by polymerase chain reaction-restriction fragment length polymorphism in blood samples from 205 NSCLC patients who had had definitive radiotherapy at one institution in November 1998–January 2005. We also tested whether the *TGF-β1* rs1982073 (T+29C) SNP affected the migration and invasion of A549 and PC9 lung cancer cells.

**Results:**

Median follow-up time for all patients was 17 months (range, 1–97 months; 39 months for patients alive at the time of analysis). Multivariate analysis showed that the *TGFβ1* rs1800469 CT/CC genotype was associated with poor OS (hazard ratio [HR] = 1.463 [95% confidence interval {CI} = 1.012–2.114], *P* = 0.043) and shorter DMFS (HR = 1.601 [95% CI = 1.042–2.459], *P = *0.032) and that the *TGFβ1* rs1982073 CT/CC genotype predicted poor DMFS (HR = 1.589 [95% CI = 1.009–2.502], *P* = 0.046) and poor brain MFS (HR = 2.567 [95% CI = 1.155–5.702], *P* = 0.021) after adjustment for age, sex, race, performance status, smoking status, tumor histology and volume, stage, receipt of concurrent radiochemotherapy, number of chemotherapy cycles, and radiation dose. Transfection with *TGFβ1*+29C (vs. +29T) stimulated the migration and invasion of A549 and PC9 cells, suggesting that TGFβ1+29C may be linked with increased metastatic potential.

**Conclusions:**

*TGFβ1* genotypes at rs1800469 and rs1982073 could be useful for predicting DMFS among patients with NSCLC treated with definitive radiation therapy. These findings require validation in larger prospective trials and thorough mechanistic studies.

## Introduction

Lung cancer remains the leading cause of cancer death, with an estimated 160,340 deaths in the United States in 2012 [1]. The current standard of care for unresectable non–small-cell lung cancer (NSCLC), by far the most common presentation at diagnosis, is radiotherapy in combination with chemotherapy. However, survival rates after such therapy remain poor because of high rates of recurrence, both local and distant. Commonly used radio(chemo)therapy regimens are also associated with significant normal tissue toxicity, including radiation pneumonitis, which can be lethal. Better treatment strategies designed according to each individual patient’s intrinsic sensitivity and risk of toxicity, the tumor’s unique expression of molecular target(s), and the risks of locoregional or distant disease recurrence are urgently needed.

One potential molecular biomarker for predicting treatment outcome after radiochemotherapy for NSCLC, in terms of both tumor response and normal tissue damage, is transforming growth factor β1 (TGFβ1). Members of the TGFβ cytokine superfamily elicit a diverse range of cellular responses, including proliferation, migration, fibrosis, apoptosis, tissue inflammation, wound repair, and angiogenesis [2]–[4]. Changes in TGFβ signaling have been reported in several types of cancer in humans, including NSCLC [5], [6]. Although TGFβ typically acts as a tumor suppressor [7], [8], as tumor development progresses, tumor cells acquire resistance to TGFβ–induced growth arrest. Of the three known TGFβ isoforms, TGFβ1 seems to be directly involved in mediating the metastatic activity of cancer cells [9]. Preclinical studies show that inhibition of TGFβ1 signaling downregulates the migration of tumor cells and suppresses the development of metastasis [10], [11]. Thus, TGFβ1 may have a dual role in cancer development, suppressing tumor growth during the initial phases of carcinogenesis but promoting tumor progression and metastasis in more advanced stages [12], [13]. Indeed, increasing evidence shows that TGFβ signaling has a role in the development of lung cancer [14], [15]. TGFβ1 has also been shown to induce the epithelial-to-mesenchymal transition, which has a crucial role in the invasion and metastasis of some types of tumor cells [16]. TGFβ has further been implicated in tumor cell sensitivity to radiation and chemotherapeutic drugs [17]–[19].

In addition to its effects on tumor cells, TGFβ1 is also involved in the response of normal tissues to radiation or radiochemotherapy. Two groups have shown preliminary evidence that changes in plasma levels of TGFβ1 during radiotherapy may predict the risk of developing radiation-induced lung toxicity [20], [21]. Strong associations have also been reported between single nucleotide polymorphisms (SNPs) in *TGFβ1* and radiation-induced fibrosis in patients with breast cancer or gynecologic cancers [22], [23] and between SNPs and radiation pneumonitis in patients with lung cancer treated with radiotherapy or radiochemotherapy [24]. Explorations of the prognostic value of *TGFβ1* SNPs in a variety of cancer types [25], [26] suggest that some *TGFβ1* genotypes predict more aggressive tumor phenotypes and adverse prognosis.

We previously assessed the relationship between three different SNPs of *TGFβ1* [rs1800469 (C–509T), rs1800471 (G915C), and rs1982073 (T+29C)] and the development of radiation pneumonitis in patients with NSCLC treated with radiochemotherapy and observed that the CT/CC genotypes of *TGFβ1* rs1982073 (T+29C) were associated with significantly lower risk of radiation pneumonitis [24]. Moreover, rs1982073 (T+29C) and rs1800469 (C-509T) have been linked with increased serum levels of TGFβ1 [26] and with the incidence of invasive breast cancer [27]; rs1800469 (C-509T) has also been linked with advanced-stage prostate cancer [26] and colon cancer [28]. On the basis of these published findings, we investigated whether these functional genetic variants of *TGFβ1* also affect tumor response and outcome in patients with inoperable NSCLC treated with definitive radiation or radiochemotherapy, assessed in terms of overall survival (OS) and distant metastasis–free survival (DMFS).

## Methods

Subjects for this retrospective analysis were selected from a large database of 740 patients with NSCLC who had been treated at The University of Texas MD Anderson Cancer Center from November 1998 through 2009. A total of 261 patients were identified as having had whole-blood samples available for analysis; all of these patients had been treated in November 1998 through January 2005. From those 261 patients, 56 were excluded: 25 who had died of other diseases or whose vital status was unknown; 16 who had died of NSCLC without information on distant metastasis or locoregional disease, 2 who had had surgery, 1 who had had small-cell lung cancer, and 12 who had had stage IV disease, leaving a total of 205 patients with complete information for the current analysis.

### Genotyping

Genomic DNA was extracted from leukocytes from whole blood samples with a DNA Blood Mini Kit (Qiagen, Valencia, CA) according to the manufacturer’s instructions. DNA purity and concentration were determined by spectrophotometric measurement of absorbance at 260 and 280 nm. SNPs in TGFβ1 were chosen that met at least two of the following three criteria: (1) reported associations with lung or other types of cancer; (2) a minor allele frequency of >5% in individuals of Caucasian descent; and (3) location in the promotor or coding region of the gene. We genotyped three such SNPs: rs1800469 (C–509T, in the promoter region), rs1800471 (G915C, in exon 1), and rs1982073 (T+29C, also in exon 1) by polymerase chain reaction (PCR) –restriction fragment length polymorphism as described elsewhere [24].

### In vitro Assessment of rs1982073 *TGFβ1+29C* or *TGFβ1+29T* Stable Transfectants

We also tested if *TGFβ1* rs1982073 (T+29C) C variant genotypes influenced the metastatic potential of lung cancer cells in vitro as follows. For these experiments, A549 and PC9 cells, both originating from human lung adenocarcinoma, were cultured in Ham’s F12 (A549) or RPMI 1640 (PC9) containing 10% fetal bovine serum and antibiotics (100 units/mL penicillin and 100 units/mL streptomycin) at 37°C with 5% CO_2_, andmedium was refreshed every 2–3 days. When the cell cultures reached about 50% confluence in fresh serum-free medium, they were transfected with a control virus or with a TGFβ1 (+29C or +29T) -overexpressing lentivirus (constructed as described below) at a multiplicity of infection of 100, incubated for 48 hours, and cultured further in Ham’s F12 (A549) or RPMI 1640 (PC9). The transfection efficiency, measured in terms of cellular expression of green fluorescent protein by fluorescence microscopy (Leica DMI4000B), was found to be >99%. The transfected cells were then tested for their invasiveness and motility as described below.

TGFβ1 cDNAs were amplified by PCR, with a cDNA library from bronchial smooth muscle cells was used as the DNA template. The primers were 5′-CCAAGCTTATGCCGCCCTCCGGGCTG-3′ and 5′-CGGAATTCTCAGCTGCACTTGCAGGAG-3′ (underlined sequences indicate the HindIII and EcoRi restriction sites). The TGFβ1 cDNAs with +29C and +29T were sent to Genechem Company (Shanghai, China) to construct the relevant lentivirus systems.

After transfection, the A549 and PC9 cells were extracted into cold lysis buffer [50 mM Tris-HCl (pH 7.5), 150 mM NaCl, 1 mM EDTA, 1 mM MgCl2, 0.5% Triton X-100, and protease inhibitor mix (1 mM PMSF, 2 µg/mL aprotinin, 1 µg/mL leupeptin, and 1 µg/mL pepstatin A)]. The cell lysates were then clarified by centrifugation at 10,000 × *g* for 20 min, the supernatants were collected, and the protein concentration of each lysate was determined with a BCA Protein Assay Kit. Equal amount of proteins in lysates were separated on 12% sodium dodecyl sulfate for polyacrylamide gel electrophoresis and transferred to polyvinylidene difluoride membranes (Bio-Rad). TGFβ1 was detected by specific antibody #3709 from Cell Signaling Technology.

Cell migration was assessed with a scratch assay and cell invasiveness with a transwell assay as follows. For the scratch assay, A549 and PC9 cells were seeded in 24-well culture plates at 2×10^5^ cells/well and incubated for 24 hours, after which the monolayer was scratched with a sterile 200-µL pipette tip, the plates were washed twice with phosphate-buffered saline, and the cells incubated with serum-free Ham’s F12 (A549) or serum-free RPMI (PC9) for 24 hours, after which images were captured by microscopy at 100× magnification and the “healing” areas were measured with a phase contrast microscope (Leica DMI4000B). For each cell type, three independent transfection experiments were performed, and all experiments were carried out in triplicate.

For the transwell assays, A549 and PC9 cells were suspended in serum-free Ham’s F12 or serum-free RPMI 1640 at a density of 3×10^5^ per mL, and 100-µL aliquots were added to the upper compartments of a 24-well transwell chamber containing polycarbonate filters with 8-µm pores and coated with 60 µL of Matrigel (1∶9 dilution; Sigma Aldrich). Five hundred-microliter aliquots of Ham’s F12 or RPMI 1640 with 10% FBS were added to the lower chambers, and the chambers were incubated for 24 hours. At that time, cells in the upper compartment were removed with a cotton swab, rinsed with PBS, and fixed in 100% methanol. Cells that had invaded through the Matrigel to the lower surface were stained with 4′,6-diamidino-2-phenylindole and quantified by counting the number of fluorescent cells in 5 random microscopic fields per filter at 400× magnification. Three independent experiments were performed.

### Statistical Analysis

In the clinical sample correlation studies, OS time was calculated from the first day of radiation treatment until death or the last known follow-up. DMFS times were calculated from the first day of radiation treatment to the first observed day of distant metastasis or the last known follow-up. Associations between the distribution of *TGFβ1* genotypes and clinical characteristics were assessed by Fisher’s exact tests or Pearson’s χ^2^ tests. Cox proportional hazards analysis was used to evaluate the influence of various *TGFβ1* genotypes on OS and DMFS, calculated as hazard ratios (HRs) with corresponding 95% confidence intervals (CIs). All HRs were adjusted for age, sex, race, Karnofsky performance status, smoking status, tumor histology, gross tumor volume, disease stage, receipt of chemotherapy or concurrent radiochemotherapy, number of cycles of chemotherapy, and radiation dose received. Kaplan-Meier analysis was used to evaluate the effect of *TGFβ1* genotype on the cumulative probability of DMFS. All reported *P* values were two-sided, and *P*<0.05 was considered to indicate statistical significance. Statistical analyses were carried out with SPSS 16.0 (SPSS Inc. Chicago, IL).

The in vitro data were expressed as means ± SD from three independent experiments (each of which had been performed in triplicate) and compared with Student’s *t* tests. *P* values of <0.05 were considered to indicate statistically significant differences.

## Results

### Clinical Sample Correlation Studies

The clinical characteristics of the 205 analyzed patients are summarized in Table 1. Patient-, disease-, and treatment-related characteristics according to *TGFβ1* polymorphisms were well balanced in terms of age, race, sex, Karnofsky performance status, tobacco use, tumor histology, gross tumor volume, disease stage, number of cycles of chemotherapy, receipt of chemotherapy vs. radiation only, and radiation dose received (not shown).

**Table 1:** Patient characteristics (n = 205).

Characteristic	No. of Patients
Sex	
Male	114 (56%)
Females	91 (44%)
Age(years)	
Median (range)	64 (35–83)
Race	
Caucasian	149 (73%)
Black	43 (21%)
Others	13 (6%)
Disease stage	
I, II	31 (15%)
III	174 (85%)
Tumor histology	
Squamous cell	68 (33%)
Adenocarcinoma	70 (34%)
NSCLC, NOS	67 (33%)
Karnofsky Performance Score	
<80	36 (18%)
80–89	115 (56%)
≥90	54 (26%)
Smoking status	
Current	52 (25%)
Former	137 (67%)
Never	16 (8%)
Treatments	
Chemoradiation	165 (80.5%)
No chemoradiation	40 (19.5%)
No. of chemotherapy cycles	
0–1	31 (15%)
2–3	70 (34%)
≥4	104 (51%)
Radiation dose (Gy)	
Median (range)	64 (50–84)

Abbreviations: NSCLC, NOS, non-small cell lung carcinoma, not otherwise specified.

The median follow-up times were 17 months for the entire group and 39 months for patients who were alive at the time of analysis (range, 1–97 months). The median OS time was 21 months. A total of 103 patients (50%) developed DM at 134 sites; 27 patients had DM at more than 1 site (23 at 2 sites and 4 at 3 sites). The sites of metastases included brain (n = 39), bone (n = 26), lung (n = 26), adrenals (n = 13), liver (n = 14), and other unspecified sites (n = 16).

**Figure 1:**
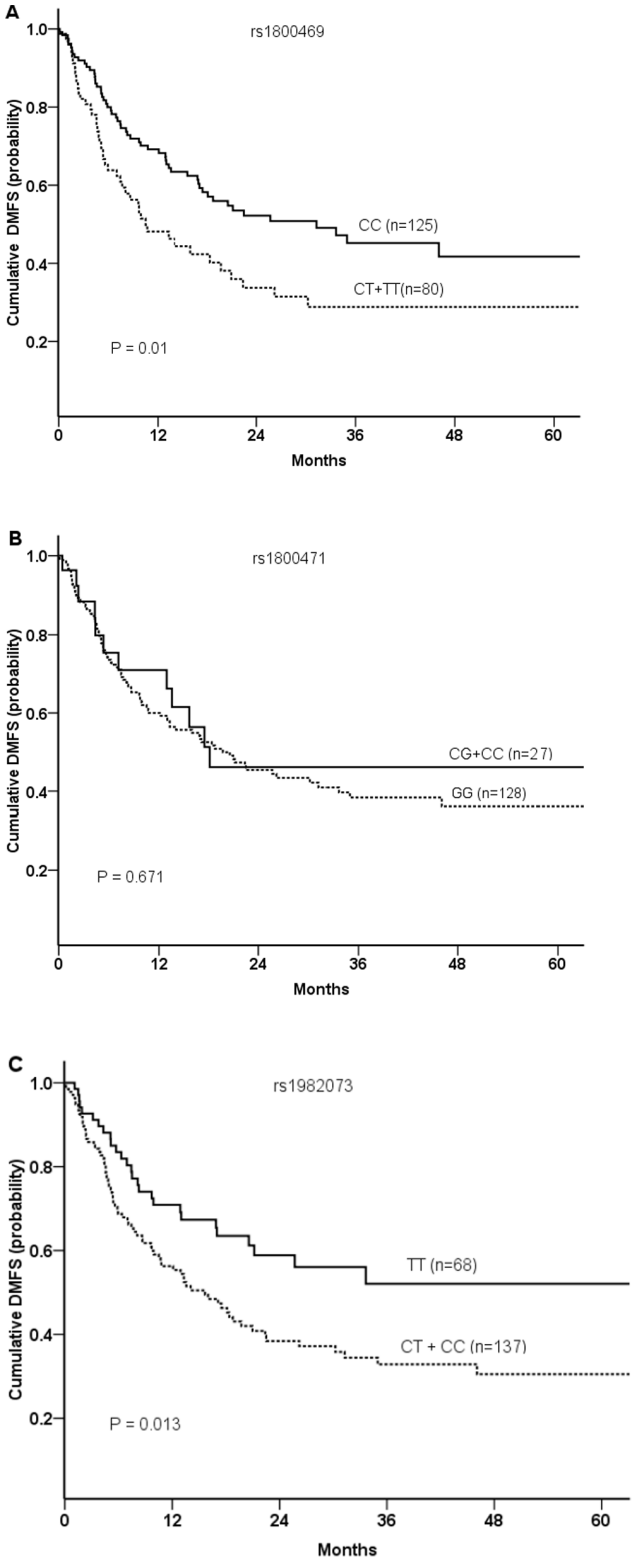
Kaplan-Meier estimates of associations between distant metastasis–free survival (DMFS) and polymorphisms of *TGF-β1* at (A) rs1800469 (C–509T), (B) rs1800471 (G915C), or (C) rs1892073 (T+29C) in patients treated with radiation or radiochemotherapy for non-small cell lung cancer. *P* values were calculated by log-rank tests.

Figure 1 illustrates the Kaplan-Meier analysis of the effect of *TGFβ1* genotype and cumulative probability of DMFS. The *TGFβ1* rs1800469 (C-509T) CT+TT genotypes were associated with significantly shorter median DMFS (11 months vs. 31 months for the CC genotype, *P* = 0.010; Fig. 1A). The *TGFβ1* rs1982073 (T+29C) CT+CC genotypes were also associated with significantly shorter median DMFS (compared with the TT genotype) (*P* = 0.013; Fig. 1C). However, no associations were found between any genotype (GG, CG, CC, or CG+CC) of the *TGFβ1* rs1800471 (G915C) SNP and DMFS (Fig. 1B).

Findings from multivariate analyses of *TGFβ1* polymorphisms and clinical outcomes adjusted for patient-, disease-, and treatment-related factors are shown in Table 2. The *TGFβ1* rs1800469 (C-509T) CT+TT genotypes were associated with increased HRs for OS and DMFS compared with the CC genotype (HR for OS = 1.463, 95% CI = 1.012–2.114, *P* = 0.043; HR for DMFS = 1.601, 95% CI = 1.042–2.459, *P* = 0.032). The *TGFβ1* rs1982073 (T+29C) CT+CC genotypes were also associated with an increased HR for DMFS compared with the CC genotype (HR = 1.589, 95% CI = 1.009–2.502, *P* = 0.046). No associations were found between any genotypes (GG, CG, CC, or CG+CC) of the *TGFβ1* rs1800471 (G915C) SNP and OS or DMFS.

**Table 2:** Multivariate analysis of *TGFβ1* polymorphisms and clinical outcome in NSCLC.

Polymorphism and Genotypes	Overall Survival			Distant Metastasis-Free Survival		
	HR	95%CI	*P*	HR	95%CI	*P*
rs1800469 (C–509T)						
CC (n = 125)	1.000			1.000		
CT+TT (n = 80)	1.463	1.012–2.114	0.043	1.601	1.042–2.459	0.032
rs1800471 (G915C)						
GG (n = 178)	1.000			1.000		
CG+CC (n = 27)	0.730	0.413–1.291	0.280	0.758	0.395–1.454	0.404
rs1982073 (T+29C)						
TT (n = 68)	1.000			1.000		
CT+CC (n = 137)	1.271	0.873–1.851	0.211	1.589	1.009–2.502	0.046

Abbreviations: NSCLC, non-small cell lung cancer; HR, hazard ratio; CI, confidence interval.

We further analyzed the association of *TGFβ1* rs1800469 (C–509T), rs1800471 (G915C), and rs1982073 (T+29C) genotypes with the organ sites in which metastases developed by using Cox proportional hazard models. The CT or TT genotypes of rs1800469 (vs. CC) and the CT or CC genotypes pf rs1982073 (vs. TT), were both associated with a twofold increase in HRs for brain, bone, and lung metastases in univariate analyses. However, in multivariate analyses, the only significant association was between rs1982073 CT or CC (vs. TT) and brain metastases (HR = 2.567 [CI = 1.155–5.702], *P* = 0.021) (Table 3).

**Table 3:** TGF-β1 genotype and brain metastasis-free survival in 102 NSCLC patients without distant metastasis and 39 NSCLC patients with brain metastasis.

Polymorphism and Genotypes	Univariate Analysis			Multivariate Analysis		
	HR*	95%CI	*P*	HR*	95%CI	*P*
rs1800469 (C–509T)						
CC (n = 125)	1.000			1.000		
CT+TT (n = 80)	1.901	1.901–3.581	0.047	1.624	0.802–3.286	0.178
rs1800471 (G915C)						
GG (n = 178)	1.000			1.000		
CG+CC (n = 27)	1.341	0.592–3.038	0.371	1.380	0.534–3.566	0.506
rs1982073 (T+29C)						
TT (n = 68)	1.000			1.000		
CT+CC (n = 137)	2.339	1.1109–4.936	0.026	2.567	1.155–5.702	0.021

* Compared with 102 patients without distant metastasis.

NSCLC, non-small cell lung cancer; HR, hazard ratio; CI, confidence interval. HRs were estimated from Cox proportional hazard models and adjusted for age, sex, race, Karnofsky performance score, smoking status, histology, disease stage, gross tumor volume, concurrent chemotherapy, number of cycles of chemotherapy, and radiation dose received.

### In vitro Findings

**Figure 2:**
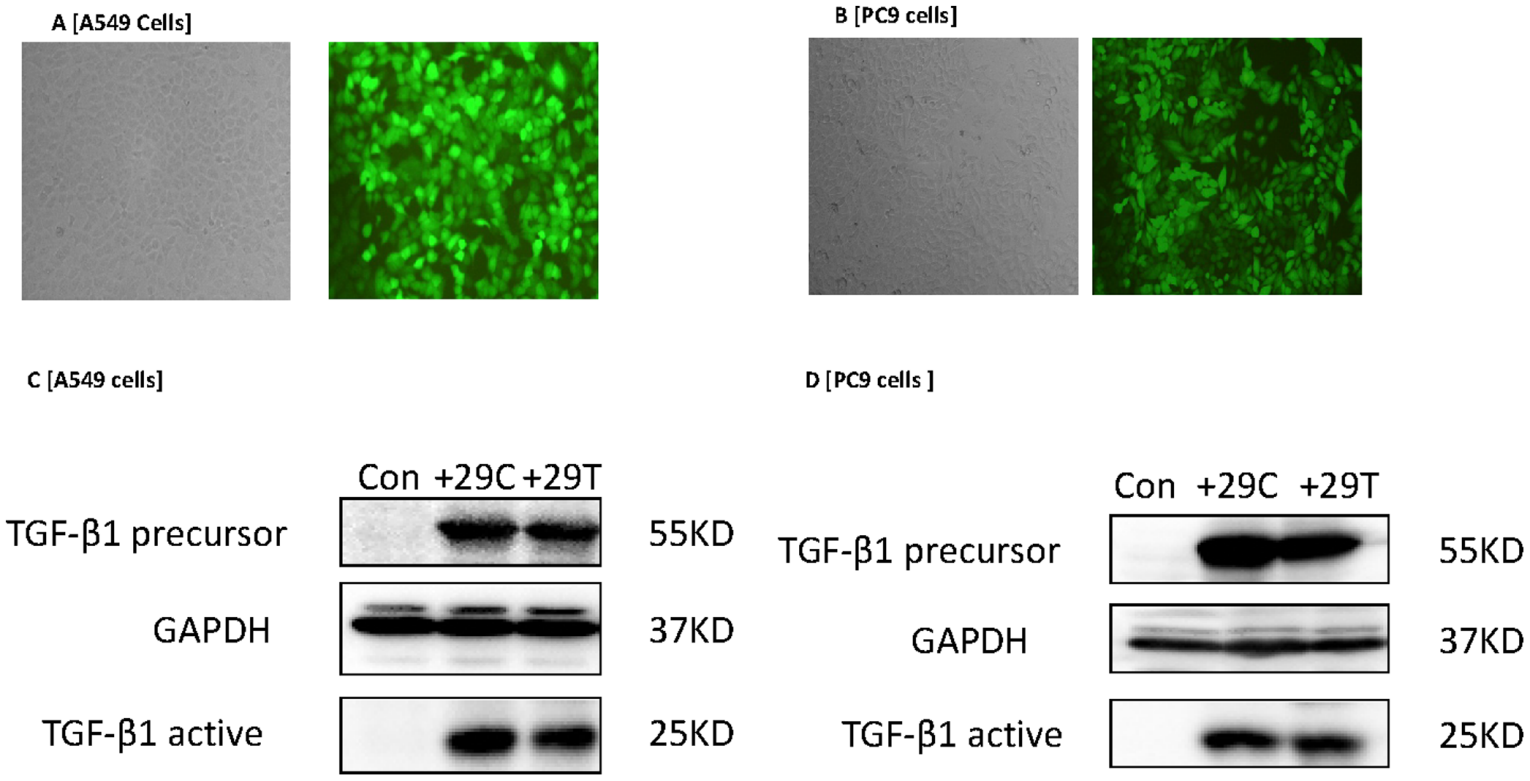
Transfection of A549 and PC9 lung cancer cell lines with lentivirus for TGFβ1 rs1892073+29C or +29T. (A,B) Fluorescence labeling indicated the transfection efficiency was >99% for both cell lines. (C,D) Western blot analysis confirmed that transfection with either +27C or +29T led to overexpression of TGFβ1 in both precursor and active forms. Con, control lentivirus construct; glyceraldehyde-3-phosphate dehydrogenase (GADPH) was used as a loading control.

**Figure 3:**
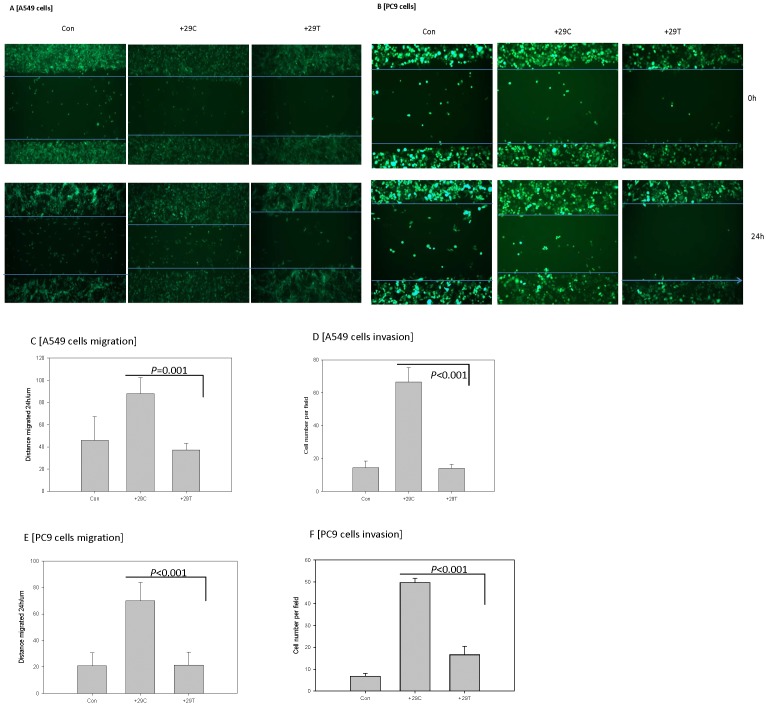
Transfection of A549 cells and PC9 cells with TGFβ1 rs1892073+29C, but not with +29T, led to enhanced motility and invasion. (A,B) Fluorescence phase-contrast microscopic images show that at 24 hours, more of the +29C transfectants migrated into a “healing” area in a scratch assay than did +29T transfectants. These findings are depicted quantitatively as the distance migrated during the scratch assay in panels C and E. (D,F) The +29C transfectants showed greater invasiveness, as indicated by numbers of cells penetrating a membrane in a transwell assay. Results are given as means from 3 independent experiments; error bars representing standard deviation.

Transfection efficiency for both A549 and PC9 lung cancer cell lines was approximately 99% (Figs. 2A,B). Transfection of A549 and PC9 cells with *TGF-β1*+29C or +29T induced the expected increases in the expression of both precursor and active forms of TGFβ1 (Figs. 2C,D). Both cell lines, upon transfection with the TGFβ1+29C construct, demonstrated increased motility (Figs. 3A,B,C,E) and increased invasion (Figs. 3D,F) relative to the +29T transfectants. These results suggest that the TGFβ1+29C genotype in both of these lung cancer cell lines induced a phenotype with increased potential for metastasis.

## Discussion

Metastasis remains a major cause of treatment failure for patients with NSCLC treated with radiotherapy, with or without chemotherapy [29], [30]. Factors that predict the biologic behavior and metastatic potential of NSCLC have been actively sought for the past 3 decades. The most significant finding from the current study is the association between variant *TGFβ1* genotypes at rs1800469 and rs1982073 and worse DMFS among patients with NSCLC given radiotherapy; this finding suggests that SNPs in *TGFβ1* genes could be used as biomarkers to prescribe personalized radiochemotherapy according to the expected pattern of recurrence before the initiation of treatment. We further validated the association of the *TGF-β1* SNP rs1982073 T+29C with enhanced migration and invasiveness of two lung cancer cell lines, A549 and PC9, in vitro.

Further analyses of our results showed a statistically higher risk of brain metastasis in patients with the *TGFβ1* rs1982073 (T+29C) C variant genotypes. Brain metastasis is a common complication of locally advanced NSCLC, with 21% to 54% of patients developing brain metastasis during the course of the disease [31], [32]. The OS time of patients with brain metastasis generally ranges from about 3 to 6 months [33]–[35], and 30%–50% of patients with brain metastases die from neurologic causes [36], [37]. Prophylactic cranial irradiation (PCI) has been shown in two randomized studies to reduce the incidence or delay the onset of brain metastasis in patients with locally advanced NSCLC after primary therapy, and use of PCI can prolong OS in such patients [38], [39]. The Radiation Therapy Oncology Group (RTOG), in an attempt to extend these results, opened a large trial (RTOG 0214) of PCI versus observation for patients with locally advanced NSCLC. In patients with stage III disease without progression of disease after therapy, PCI decreased the rate of brain metastasis but did not improve OS or disease-free survival [40]. Ideally, one would prescribe PCI only for patients at high risk of developing brain metastases. However, so far, no reliable way has been found to identify which patients will develop brain metastasis and which will not–hence the intense search for biomarkers. Therefore, our finding that *TGFβ1* rs1982073 (T+29C) C variant genotypes were associated with brain metastasis is of particular importance in the management of NSCLC, because this SNP might serve as a biomarker to identify patients who would benefit from PCI.

The possibility of using *TGFβ1* SNPs as predictive biomarkers in other kinds of cancer has been studied by others. In one such study, the *TGFβ1* rs1800469 C–509T homozygous TT genotype was found to be associated with an increased risk of having an aggressive form of prostate cancer, but the rs1982073 T+29C (T+29C) C variant genotypes were not [26]. In contrast, another group showed that patients with breast cancer who carried the T+29C C allele had lower 5-year disease-free survival rates than did those with the TT genotype [25]. Other evidence is emerging that SNPs in some other genes may predict outcome in NSCLC. For example, genetic aberrations in genes for matrix metalloproteinase (MMP) -3 and MMP-9 or the x-ray repair protein XRCC1 have been reported to influence the clinical behavior and risk of metastasis of lung cancer [41]–[43]. These biomarkers have yet to be widely translated into clinical practice. Our findings are in line with these reports: DM was a major pattern of failure in our study (DMFS rate, 50.2%), and variant genotypes at *TGFβ1* rs1800469 (C-509T) and *TGFβ1* rs1982073 (T+29C) seemed to be associated with shorter DMFS times. If validated in larger studies, our findings could serve as the basis for using these genotypes as a prognostic marker of clinical outcome, particularly predisposition to brain metastasis, in patients with NSCLC.

The mechanistic basis of the findings from the current study needs further investigation. Some investigators have suggested that TGβ1 protein levels might independently predict survival in patients with adenocarcinoma of the lung [18], [44]–[46]. In those studies, TGFβ1 expression in primary lung cancer tissues was higher among patients with pulmonary metastases than that among patients without such metastases. Earlier reports suggested that polymorphisms in the signal sequence at +915 [47], C–509T [48], and T+29C (encoding Leu10Pro) in the *TGFβ1* gene were associated with increased TGFβ1 production. Those studies may shed light on our finding that variant genotypes at *TGFβ1* rs1800469 (C-509T) and *TGFβ1* rs1982073 (T+29C) were associated with shorter DMFS times in patients with NSCLC treated with definitive radiation therapy.

In our study, having the *TGFβ1* rs1800469 (C-509T) CT or TT genotypes seemed in univariate analysis to be associated with shorter OS, and no associations were found between OS and *TGFβ1* rs1982073 (T+29C) genotypes. This finding is inconsistent with a report by Mu et al. [49] showing that patients with early-stage breast cancer and the T/T genotype or high intratumoral TGFβ1 levels had shorter OS times than did those without T/T or with low intratumorial TGFβ1 levels. However, this association was different for patients with late-stage disease: for those patients, having the T/T genotype was associated with lower risk of disease recurrence. These findings indicate that *TGFB1* has complex roles in breast cancer progression, which supports the findings of other studies that *TGFβ1* has conflicting effects on tumor growth and metastasis.

Our study had several limitations; its single-institution and retrospective nature require that its findings be validated in larger, preferably multi-institutional prospective trials. Another limitation was that we did not measure TGFβ1 protein levels in plasma before, during, or after radiation therapy, and so we could not determine if having the *TGFβ1* rs1982073 (T+29C) CT genotype influenced the expression of TGFβ1 protein levels in vivo, nor could we determine whether the risk of DM correlates with changes in this variable before or after treatment. Thus clarification is still needed regarding the relationships among polymorphisms in *TGFβ1* and TGFβ1 protein levels in plasma and prognosis in patients with NSCLC. Finally, our research was limited to patients who received definitive radiation therapy. Whether the *TGFβ1* rs1982073 variant genotypes also predict outcome among patients who undergo surgery or other non-radiation-based therapies remains unclear.

Our previous work [24] showed that having the CT or CC variants in *TGFβ1* rs1982073 was associated with lower risk of severe (grade ≥3) radiation pneumonitis, an observation suggesting that radiation dose intensification to increase locoregional tumor control in patients with this genotype would be feasible. However, the results we report here showed that patients with those genotypes were at increased risk of DM, specifically brain metastases, an observation suggesting that systemic treatment and PCI are indicated. The results from our study suggest, for the first time, that the *TGFβ1* rs1982073 variant genotypes may be useful as a genetic biomarker to help physicians tailor the treatment according to the patient’s risk of radiation pneumonitis and DM. As such, additional studies are needed to validate this finding and to investigate the mechanisms by which *TGFβ1* polymorphisms influence the course of lung cancer development and progression. These kinds of studies will help to provide a better understanding of the cancer microenvironment and metastasis and may be instrumental in tailoring treatment for NSCLC.
